# Smart chemometrics-assisted spectrophotometric methods for efficient resolution and simultaneous determination of paracetamol, caffeine, drotaverine HCl along with three of their corresponding related impurities

**DOI:** 10.1186/s13065-023-01036-8

**Published:** 2023-10-05

**Authors:** Samia A. Tawfik, Maha A. Hegazy, Nariman A. El-Ragehy, Ghada A. Sedik

**Affiliations:** Department of Analytical Chemistry, Faculty of Pharmacy, Cairo University, Kasr El-Aini St., Cairo, 11562 Egypt

**Keywords:** Caffeine, Drotaverine HCl, Homoveratric acid, Impurities, *P*-aminophenol, Paracetamol, PCR, PLS, siPLS, Theophylline

## Abstract

Three novel, simple and accurate multivariate spectrophotometric assisted mathematical techniques were developed for determination of paracetamol, caffeine, drotaverine HCl and their related impurities. The used multivariate algorithms are principal component regression (PCR), partial least squares (PLS), and synergy intervals partial least squares (siPLS). Linearity of the suggested methods was found to be (1.00–14.60, 1.40–7.00, 1.40–3.80, 1.00–3.00, 1.50–3.50 and 2.50–4.50 µg/mL) for paracetamol, caffeine, drotaverine HCl, and their related impurities; *p*-aminophenol, theophylline and homoveratric acid, correspondingly. The presented methods were effectively implemented in the determination of the cited compounds in their laboratory prepared mixtures. Commercially available tablet preparation was also analyzed using the applied methods where no impurities were detected and without interference from tablet additives. Moreover, statistical analysis did not reveal any noticeable differences between the obtained results and those acquired from the reported method in terms of accuracy and precision. The developed multivariate algorithms were validated by means of internal and external validation sets. The obtained results showed the siPLS algorithm’s superiority to PCR and PLS according to the values of correlation coefficient values (r) and the lowest root mean square error of prediction (RMSEP). The combination of four subintervals [10, 12, 14, and 17] produced the highest efficiency model. Furthermore, these methods may be an applicable substitute to HPLC ones in quality control laboratories during rush of analyses where several samples have to be analyzed in a short time.

**Supplementary Information:**

The online version contains supplementary material available at 10.1186/s13065-023-01036-8.

## Introduction

Paracetamol (PAR), *N*-(4-hydroxyphenyl)acetamide [1], is considered to be the most frequently used over-the-counter medicine worldwide [2]. It is described for the treatment of many symptoms such as headache, cold, fever, muscle aches, and toothache [3]. Caffeine (CAF), 1,3,7-trimethyl-3,7-dihydro-1*H*-purine-2,6-dione [1], is a central nervous system stimulating pharmaceutical prescribed to treat tiredness, drowsiness, and to potentiate the effect of some pain remedies [3]. Drotaverine HCl (DRO), 1-[(3,4-diethoxyphenyl)methylidene]-6,7-diethoxy-1,2,3,4-tetrahydroisoquinoline, [4] is a non-official spasmolytic pharmaceutical that acts via inhibition of phosphodiesterase-4 enzyme. It is utilized mainly to cure gastrointestinal, biliary and vasomotor disorders caused by muscle spasms [5]. The three pharmaceuticals are combined in Petro® tablets which are used to treat some symptoms as fever, and renal or biliary colic spasms [6].

In the last decades, impurity profiling has become an essential part of the pharmaceuticals’ overall industry. Their existence, even in tiny quantities, can affect not only drug efficacy but also drug safety [7]. Several regulatory authorities like the United States Food and Drug Administration (FDA) and the International Council for Harmonisation (ICH) shed their light on the purity requirements as well as impurity detection in active pharmaceutical ingredients [8]. Analytical researchers face a significant challenge in both qualitative and quantitative analysis of impurities to meet acceptable standards [9]. PAR has eleven different impurities listed in British Pharmacopoeia (BP) [1]. *P***-**aminophenol (PAP) is reported to be an official impurity for PAR in the British Pharmacopoeia (BP) [1] besides the United States Pharmacopeia (USP) [10]. It is the main co-existing impurity of PAR in pharmaceutical preparations that was originated from either synthesis or degradation of PAR [11]. It is noteworthy to mention that it has also a nephrotoxic effect [12]. CAF is an official drug that has six mentioned impurities in BP [1]. Theophylline (THEO),1,3-Dimethyl-3,7-dihydro-1*H*-purine-2,6-dione,is cited as a CAF impurity A in BP [1]. It is also recommended as a treatment for reversible airways obstruction [13]. In addition; THEO has been reported to induce tachycardia and tachyarrhythmia in persons when it was taken in high doses with CAF [14]. It shows toxicity symptoms in high concentration serum level (> 25.0 μg/mL) that makes its determination is crucial [15]. DRO is reported to have four known impurities produced as a result of DRO synthesis or degradation [16]. Homoveratric acid (HVA), 3,4-dimethoxyphenyl acetic acid, has been identified to be one of these impurities [16]. A review of the existing literature on determining PAR, CAF and DRO in their mixture indicated two different spectrophotometric chemometric methods [6, 17], four HPLC methods [17–20] and one TLC-densitometric method [6]. However, none of these chemometric methods have considered the determination of the related impurities together with the studied pharmaceuticals. Consequently, the objective of the present study is to develop and then validate simple, selective and economical chemometric methods (PCR, PLS, and siPLS) for the quantitative determination of PAR, CAF and DRO along with their related impurities without any prior separation steps.

## Experimental

### Instruments

Spectrophotometric measurements were done using Shimadzu 1650 UV-PC spectrophotometer (USA), by two identical 1.00 cm quartz cells. Scans have been taken in the range of 200.0–400.0 nm at 0.1 nm interval. The used wavelength scanning speed was 2800 nm/min.

### ***Software***

Shimadzu UV-Probe 2.32 system software was used to automatically generate the spectra. Matlab® version 9.4.0, Mathworks Inc., 2018 was used along with PLS_ Toolbox 2.1 for all data calculations and analysis. The iToolbox was also used for siPLS model construction.

### Materials

#### Pure standards

Standard materials of PAR, CAF and THEO were generously donated by the Egyptian International Pharmaceutical Industries Company (EIPICO), Egypt. The purities were checked using official HPLC method and found to be 101.04 ± 0.772, 100.63 ± 1.559 and 99.70 ± 1.046, correspondingly [10]. DRO was kindly offered from Alfacure pharmaceuticals, Egypt. Its purity was checked and found to be 99.86 ± 1.548 using reported UPLC method [16]. PAP and HVA were purchased from Sigma Aldrich, Germany with a checked respective purity of 99.87 ± 1.064 and 101.09 ± 0.905. Their purities were examined according to the official HPLC method [10] and the reported HPLC method [16], respectively.

#### Pharmaceutical formulation

Petro® tablets, claimed to contain 400 mg of PAR, 60 mg of CAF and 40 mg of DRO. It is manufactured by Alfacure pharmaceuticals, Egypt and was bought from pharmacies.

#### Chemicals and solvents

Potassium dihydrogen phosphate and methanol (Sigma-Aldrich, Steinheim, Germany), double distilled water (Alfa Aesar, Cairo, Egypt).

### Standard solutions

#### Stock standard solutions

Stock standard solutions of PAR, CAF, DRO (1.00 mg/mL), PAP, THEO and HVA (500.00 µg/mL) were prepared in six separate 100-mL volumetric flasks. They were prepared by accurately and separately weighing 100.00 mg of PAR, CAF, DRO and 50.00 mg of PAP, THEO and HVA and dissolving in enough volume of methanol then the volumes were completed to the mark using methanol.

#### Working standard solutions

Working standard solutions of PAR, CAF, DRO (100.00 µg/mL), PAP, THEO and HVA (50.00 µg/mL) were prepared in six different 100-mL volumetric flasks. They were prepared through accurately measuring and transferring 10.0 mL from their respective stock standard solutions, separately into the volumetric flasks. The volumes were then completed to the mark with methanol.

### Procedures

#### Construction of calibration model

A five-level, six-factor design was implemented via five different concentration levels taking codes from − 2 to + 2 per each compound. There are five mixtures at each concentration level for each component, leading to 25 mixtures [21]. Randomly, 20 mixtures were selected to be a calibration (training) set. This set was prepared by accurately transferring various aliquots from each of the six components’ working standard solutions into a series of 25-mL volumetric flasks. The concentration ranges of PAR, CAF, DRO PAP, THEO and HVA in the produced mixtures were 1.00–14.60, 1.40–7.00, 1.40–3.80, 1.00–3.00, 1.50–3.50 and 2.50–4.50 µg/mL, respectively. The central level of the design is 7.80 µg/mL, 4.20 µg/mL, 2.60 µg/mL, 2.00 µg/mL, 2.50 µg/mL and 3.50 µg/mL for PAR, CAF, DRO, PAP, THEO and HVA, correspondingly. Table 1 represents the concentration design matrix including calibration and validation sets. The absorption spectra of these solutions were scanned in the range of 200.0–400.0 nm and the data points were then exported to Matlab® for further manipulation using PLS Toolbox and iToolbox [22].

**Table 1:** Concentration of the six studied components in the calibration and validation sets

Samples	Concentration (µg/mL)					
	PAR	CAF	DRO	PAP	THEO	HVA
1	7.80	4.20	2.60	2.00	2.50	3.50
2	7.80	1.40	2.00	1.00	3.50	4.50
3	1.00	2.80	1.40	3.00	3.50	3.50
4	4.40	1.40	3.80	3.00	2.50	3.00
5	1.00	7.00	3.80	2.00	2.00	4.50
6	14.60	7.00	2.60	1.50	3.50	3.00
7	14.60	4.20	2.00	3.00	2.00	4.00
8	7.80	2.80	3.80	1.50	3.00	4.00
9	4.40	7.00	2.00	2.50	3.00	3.50
*10*	*14.60*	*2.80*	*3.20*	*2.50*	*2.50*	*4.50*
11	4.40	5.60	3.20	2.00	3.50	4.00
12	11.20	5.60	2.60	3.00	3.00	4.50
*13*	*11.20*	*4.20*	*3.80*	*2.50*	*3.50*	*2.50*
14	7.80	7.00	3.20	3.00	1.50	2.50
15	14.60	5.60	3.80	1.00	1.50	3.50
16	11.20	7.00	1.40	1.00	2.50	4.50
17	14.60	1.40	1.40	2.00	3.00	2.50
18	1.00	1.40	2.60	2.50	1.50	4.00
*19*	*1.00*	*4.20*	*3.20*	*1.00*	*3.00*	*3.00*
*20*	*7.80*	*5.60*	*1.40*	*2.50*	*2.00*	*3.00*
21	11.20	1.40	3.20	1.50	2.00	3.50
22	1.00	5.60	2.00	1.50	2.50	2.50
*23*	*11.20*	*2.80*	*2.00*	*2.00*	*1.50*	*3.00*
24	4.40	2.80	2.60	1.00	2.00	2.50
25	4.40	4.20	1.40	1.50	1.50	4.50

The italics samples are those selected for external validation

#### Validation of calibration models

The developed calibration approaches were submitted to internal and external validation. Firstly, internal validation (cross validation) was tried by means of random subsets, leave one out, venetian blinds and contiguous block where the results were improved using random subsets. Moreover, an external validation set was randomly selected. The set consists of five mixtures having various ratios of the cited components. Different aliquots were accurately transferred from the working standard solutions of the studied components into 25-mL volumetric flasks. Then, the volumes were completed with methanol to the mark. The spectra of these solutions were scanned from 200.0–400.0 nm and the data was then utilized to measure the predictivity of the constructed models through determination the six components’ concentration in each mixture.

#### Assay of pharmaceutical formulation (Petro® tablets) and application of standard addition technique

Ten tablets were accurately weighed, finely powdered and then mixed properly. A quantity equal to average weight of one tablet was weighed and transferred accurately into a 100-mL volumetric flask. After that, 60 mL methanol was added to dissolve the powder and the solution was sonicated for about 30 min. The volume was then completed to the mark with the same solvent and thoroughly mixed. The obtained solution was filtered then, an accurately measured aliquot (6.25 mL) from the obtained filtrate was transferred into a 50-mL volumetric flask, and diluted with methanol to the mark. An aliquot of 0.70 mL from the prepared solution was properly transferred into a 25-mL volumetric flask then the volume was completed to the mark with methanol and mixed well. The final solution’s concentration is claimed to be 14 µg/mL PAR, 2.1 µg/mL CAF and 1.4 µg/mL DRO. The absorption spectrum of this solution was scanned in the range of 210.0–335.0 nm with an interval of 0.2 nm. The concentration of the studied pharmaceuticals and their corresponding impurities were calculated through the developed calibration models. The validity of the adopted methods was assessed by applying standard addition technique through spiking the pharmaceutical formulation with known masses of standard compounds powders of PAR, CAF, DRO, PAP, THEO and HVA. The recoveries of the added standards were then calculated after applying the developed methods.

## Results and discussion

Spectrophotometry is one of the simplest, rapid and cost-effective techniques compared to expensive chromatographic ones. Therefore, it can be used for the assay of several mixtures with high level of precision and accuracy [23]. To continue our aim of simplicity, spectrophotometry was then combined with advanced multivariate methods to allow simultaneous resolution and determination of the aforementioned pharmaceuticals along with their related impurities despite of their overlapped spectral signals. Chemometrics is science of acquiring useful information from analytical numerical data [24, 25]. In this contribution, three different multivariate calibration models namely; PCR, PLS and siPLS were developed. PCR and PLS models were frequently applied in quantitative pharmaceuticals analysis to get specific information from more general data [26]. In addition, further advanced chemometric algorithms (such as; siPLS) are recently introduced to be applied to all types of numerical data sets with advantage of signal selection to achieve better performance [27].

**Fig. 1:**
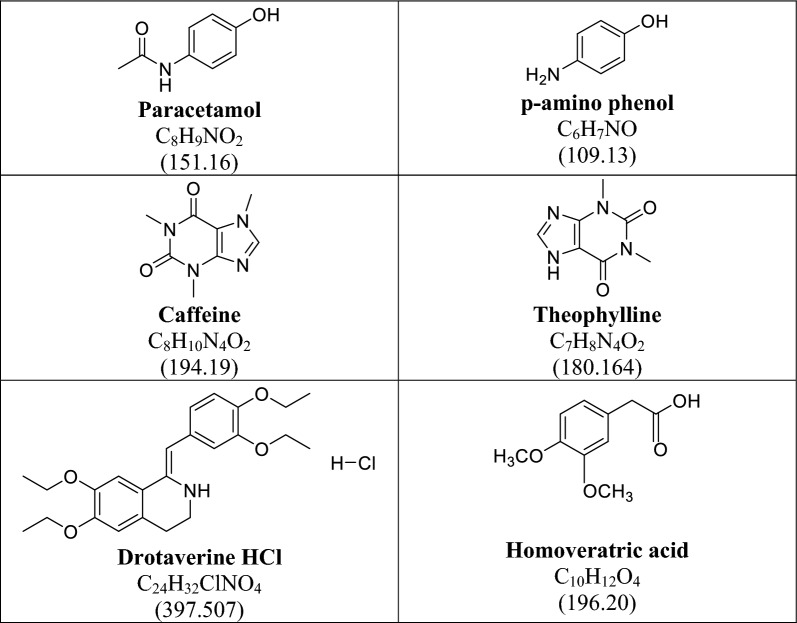
Chemical structure, molecular formula and molar mass in grams for the studied compounds

**Fig. 2:**
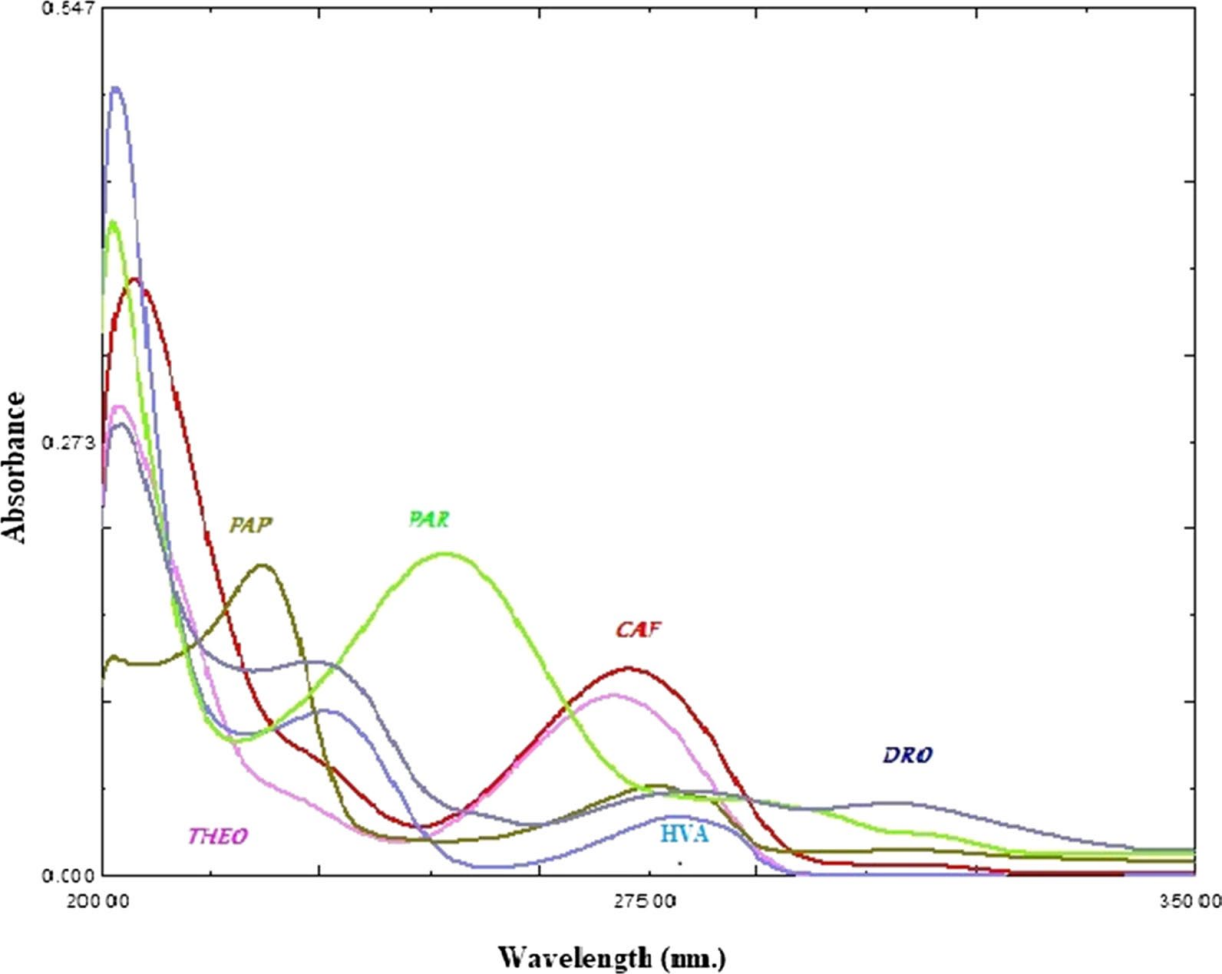
Absorption spectra of 2.00 μg/mL paracetamol (PAR), 2.00 μg/mL caffeine (CAF), 2.00 μg/mL, drotaverine HCl (DRO), 2.00 μg/mL *p*-aminophenol (PAP), 2.00 μg/mL theophylline (THEO) and 2.50 μg/mL homoveratric acid (HVA) using methanol as a solvent

Nowadays, impurity profiling has become mandatory in the pharmaceutical research. It includes isolation, characterization then quantitative determination of these impurities [8]. Presence of impurities in pharmaceutical formulations can be occurred due to the active pharmaceutical ingredients, inert additives, the formulation and also packaging processes [28]. Therefore, the authors focused on determination of the studied mixture; PAR, CAF and DRO in presence of their potential impurities; PAP, THEO and HVA. The chemical structures and molecular weights of the studied compounds are shown in Fig. 1. The absorption spectra of the six studied components using methanol as a solvent are represented in Fig. 2. The spectra of the six compounds are severely overlapped which makes their determination in direct way is impossible. This type of spectral similarity and overlapping cannot be resolved by means of univariate spectrophotometric methods. Hence, multivariate calibration methods can be a worthy way since the data submitted to analysis are able of quickly and accurately resolving and determining each of the six components in a short time [24]. A six factor, five-level calibration model was designed to prepare mixtures of PAR, CAF, DRO, PAP, THEO and HVA. In order to construct the regression models, a training set of 20 mixtures was randomly chosen, and an external validation set of the remaining five mixtures was employed, as shown in Table 1.

The scanning range for the prepared samples was 210.0–335.0 nm and the spectral data acquisition was taken with 0.2 nm intervals, therefore generating 626 data points for each spectrum. The resulting spectral data matrix comprises 25 rows representing 25 samples and 626 columns representing the wavelengths (25 × 626).

### Principal component regression (PCR) and partial least squares (PLS)

PCR and PLS are multivariate calibration approaches based on principal component analysis. They are considered factor–based full spectrum algorithms that allow rapid and simple quantitative determination of many complex pharmaceutical mixtures in different matrices [29]. In contrast to PCR, PLS analyses both the concentration and absorbance matrices before extracting components known as latent variables (LVs) [30].

For applying PCR and PLS, the raw data of 20 calibration mixtures were subjected to auto scaling and mean centering as a preprocessing step but both types of preprocessing don’t work. Several cross validation methods were tried such as leave one out, venetian blinds, contiguous block and random subsets [31]. The best results were found utilizing random subsets with four splits and five iterations to be used as a cross validation method. The full data spectra couldn’t make an efficient determination of the complex six component system. Choosing wavelength region with the most useful interval can enhance the prediction ability by recognizing the most relevant band in the examined spectra. As a consequence, the spectral band 210.0–335.0 nm, with interval of 0.2 nm was found to be more efficient with fewer LVs number. The root mean square error of cross validation (RMSECV) has been calculated by means of cross validation method (random subsets) to select the optimum factors number.

For PCR and PLS regression models, choice of optimum factors’ number is a crucial step before calibration models construction. This can be attributed to the fact that if the chosen number of factors was higher than required, extra noise will be introduced to the data. Alternately, if the selected number was too small, valuable data that may be required for the calibration model might be discarded [32]. The optimal number of latent variables (LVs) in the data was found to be 9 and 8 in PCR and PLS models, in order. The excessive number of LVs is attributed to the high similarity in the spectra between each pharmaceutical and its corresponding impurity which leads to presence of multicollinearity [33]. The residual error values were calculated for each component concentration and the graphs were constructed using PCR and PLS models, Additional file 1: Figures S1, S2, respectively. The performance characteristics of the applied regression models were evaluated via an external validation set consisting of five different mixtures. The percentage recoveries for the studied components were calculated showing satisfactory results, Additional file 1: Table S1. Graphs were constructed by plotting the predicted concentrations for each compound by each of the developed models versus its true concentrations. Table 2 displays the statistical and linear regression parameters of the validation set. It shows that the slope approached one and the intercept approached zero upon applying PCR and PLS regression models which indicate good prediction of the calibration models.

**Table 2:** Statistical and linear regression parameters for the validation set using PCR and PLS models

Parameters	PCR						PLS					
	PAR	CAF	DRO	PAP	THEO	HVA	PAR	CAF	DRO	PAP	THEO	HVA
Mean recovery %	99.95	99.52	100.59	99.29	100.59	99.52	100.15	99.67	100.84	99.54	100.69	99.97
SD	1.362	3.614	4.698	2.207	2.438	1.394	1.216	3.404	1.665	2.471	2.2468	1.376
RSD %	1.362	3.631	4.671	2.223	2.424	1.401	1.214	3.415	1.651	2.482	2.231	1.376
RMSEP	0.11851	0.12493	0.12992	0.04278	0.06930	0.04034	0.12922	0.11551	0.05668	0.04232	0.05858	0.04006
Correlation coefficient (r)	0.9976	0.9910	0.9795	0.9966	0.9921	0.9970	0.9976	0.9930	0.9984	0.9966	0.9935	0.9973
Slope	1.0045	0.9304	0.9617	0.9720	1.0245	0.9950	0.9759	0.9295	1.0353	0.9666	0.9877	0.9707
Intercept	− 0.0475	0.2326	0.1145	0.0371	− 0.0435	0.0003	0.2772	0.2419	− 0.0656	0.0518	0.0433	0.0896
LOD^a^	0.077	0.416	0.524	0.078	0.248	0.148	0.384	0.365	0.129	0.092	0.227	0.147
LOQ^a^	0.234	1.257	1.289	0.238	0.752	0.449	1.064	1.106	0.390	0.278	0.688	0.447

^a^Calculated from equation [LOD (limit of detection) = 3.3 (SD/S), LOQ (limit of quantification) = 10 (SD/S); where SD is the standard deviation of regression residuals and S is the slope of the calibration curves

### siPLS model

**Fig. 3:**
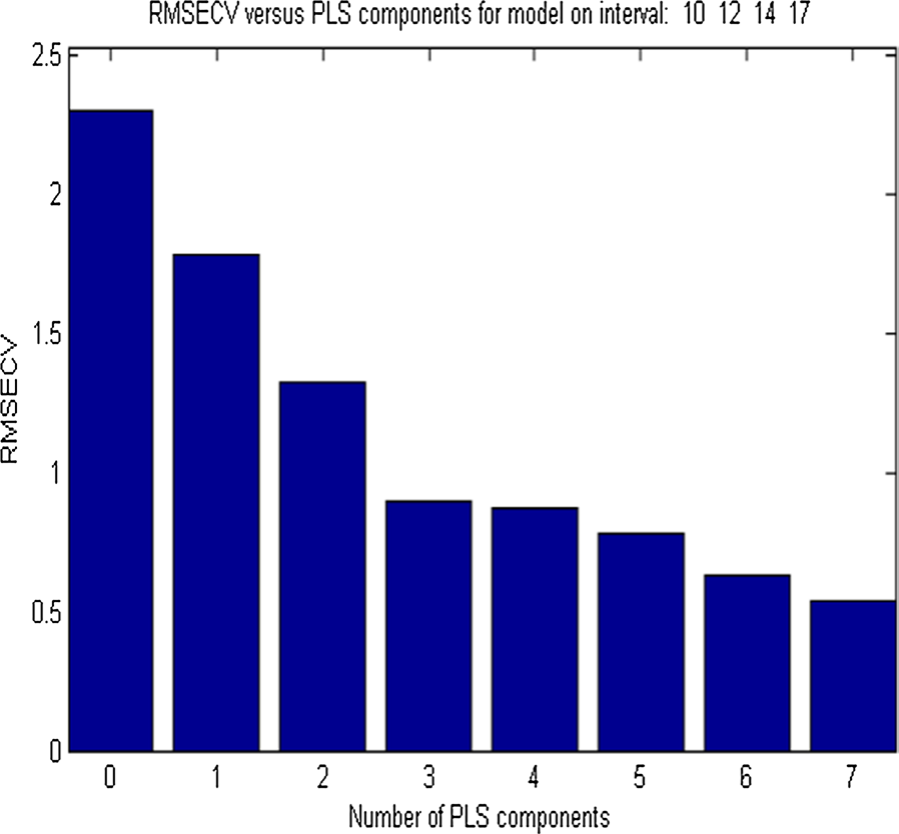
RMSECV against PLS components for siPLS model on interval of [10:12:14:17]

**Fig. 4:**
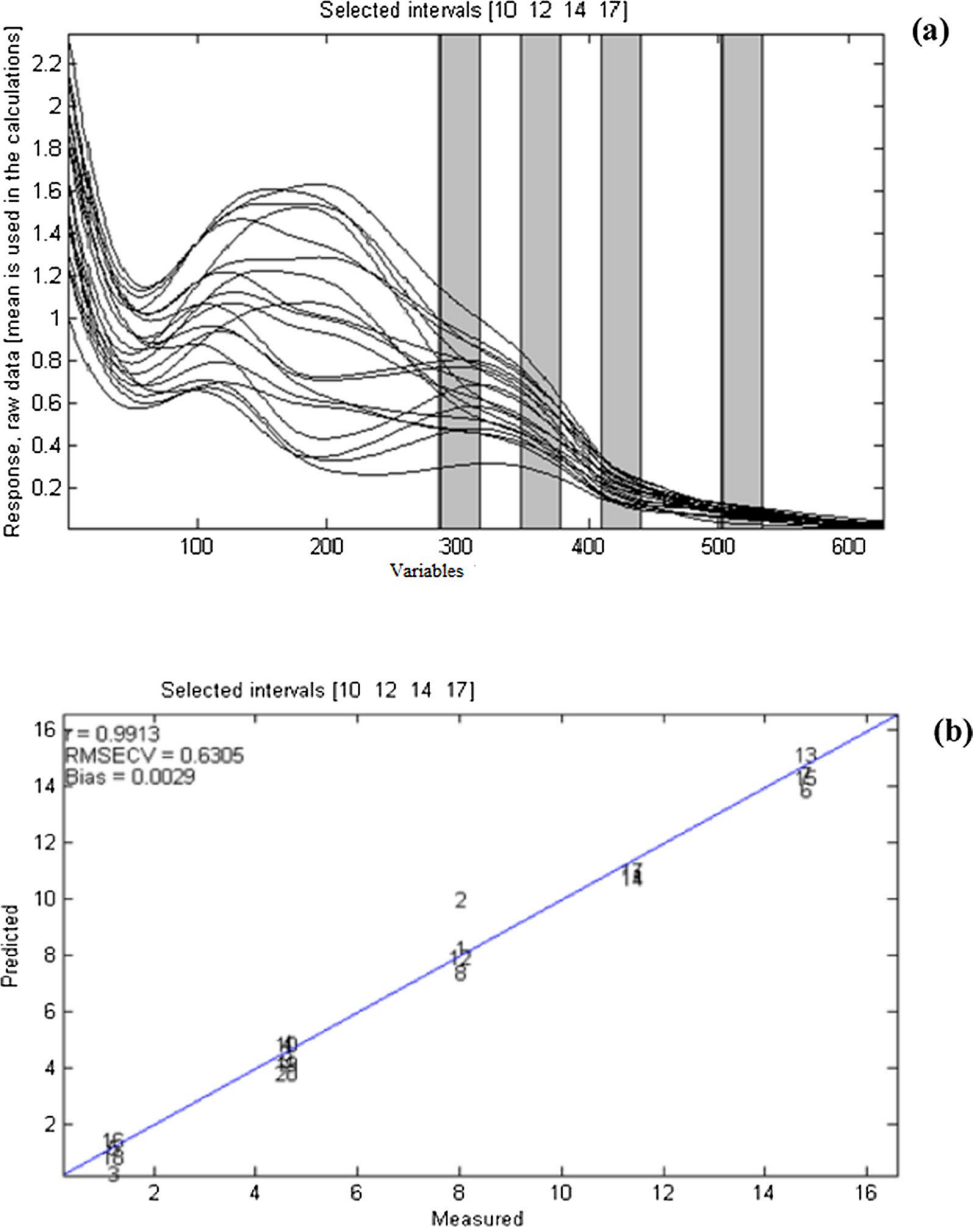
Spectral regions selected to build the models and results: **a** siPLS model by combination of subintervals [10:12:14:17] for quantification. **b** Average content of the six components (μg/mL) against the predicted values by cross-validation for the siPLS model with 7 LVs

siPLS is a variable selection method that relies on the division of data set into equidistant intervals and calculating all probable siPLS models by making combinations of two, three, or four intervals. Enormous models are produced according to intervals number and the number of selected combined intervals. After that, the obtained results are automatically displayed as the PLS components number and interval combinations. The RMSECV values for the excellent models are also calculated. It is worth mentioning that these values depend basically on the intervals number and intervals combinations. The siPLS model was applied to the studied mixture in order to find the best informative regions, which lead to improve components prediction ability, minimize interference, and decrease latent variables number when compared to PCR and PLS. Many combinations of equidistant intervals were produced and tested. For each combination of two, three, and four intervals, the PLS regression model was applied. The combination of these four subintervals [10:12:14:17] with (266.3–272.5 nm, 278.7–285.0 nm, 291.2–297.5 nm & 310–316.3 nm) as corresponding selected wavelength regions have produced the best results, with 7 latent variables as shown in Figs. 3 and 4. The error associated with each studied compound was calculated as shown in Additional file 1: Table S2.

**Fig. 5:**
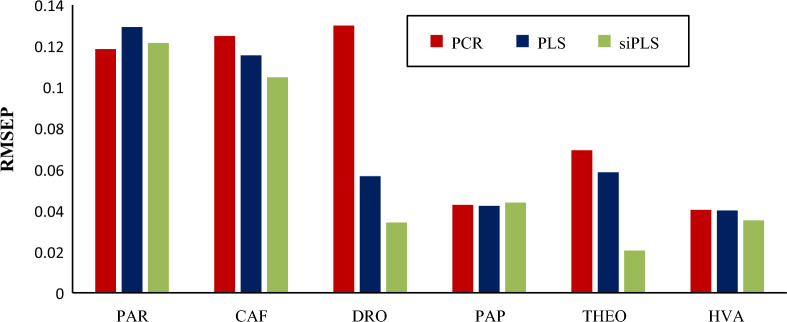
Comparison of RMSEP of paracetamol (PAR), caffeine (CAF), drotaverine HCl (DRO) along with their related impurities; *p*-aminophenol, (PAP) theophylline (THEO) and homoveratric acid (HVA) between the three proposed chemometric methods

Table 3 represents the RMSE results of siPLS model showing that [10:12:14:17] subintervals combination has the lowest RMSE value of 0.5423. Graphs were constructed relating the residual error values and the concentrations for the six studied compounds, Additional file 1: Figure S3. The percentage recoveries of the studied concentrations in the validation set mixtures are listed in Additional file 1: Table S3 showing acceptable results. The statistical and linear regression parameters of the validation set are presented in Table 4. Comparison of RMSEP values for the three aforementioned models of the six studied components is shown in Fig. 5 ensuring the excellence of siPLS to the applied PCR and PLS models.

**Table 3:** Statistical results of siPLS model for the six studied components

PLS component	Intervals^a^	RMSE
*7*	*[10 12 14 17]*	*0.5423*
7	[9 12 14 17]	0.5470
7	[10 11 14 17]	0.5480
7	[10 13 14 18]	0.5537
7	[10 12 14 18]	0.5594
7	[10 13 14 17]	0.5639
7	[9 13 14 16]	0.5649
7	[10 11 14 18]	0.5664
7	[9 12 14 16]	0.5707
7	[2 5 18 19]	0.5730

The italic row represents the selected intervals

^a^Original number of intervals is 20

**Table 4:** Statistical and linear regression parameters of the validation set using siPLS model

Parameters	siPLS [10 12 14 17]					
	PAR	CAF	DRO	PAP	THEO	HVA
Mean recovery %	100.66	98.03	101.20	98.65	100.56	100.33
SD	1.075	1.280	1.149	1.466	0.954	0.957
RSD %	1.068	1.3063	1.135	1.486	0.949	0.954
RMSEP	0.12149	0.10483	0.03419	0.04382	0.02054	0.03517
Correlation coefficient (r)	0.9985	0.9996	0.9994	0.9993	0.9998	0.9998
Slope	0.9776	0.9467	0.9935	0.9616	0.9765	1.0445
Intercept	0.3112	0.1226	0.0440	0.0459	0.067	− 0.1267
LOD^a^	0.353	0.081	0.078	0.056	0.033	0.032
LOQ^a^	1.070	0.245	0.237	0.169	0.100	0.096

^a^Calculated from equation [LOD (limit of detection) = 3.3 (SD/S), LOQ (limit of quantification) = 10 (SD/S); where SD is the standard deviation of regression residuals and S is the slope of the calibration curves

### Application on pharmaceutical dosage form and application of standard addition technique

The developed multivariate approaches were applied to quantitatively determine PAR, CAF and DRO in Petro® tablets. Table 5 shows good results within the accepted formulation range. None of the studied impurities were detected in the pharmaceutical formulation. The validity of the three suggested models was evaluated by applying the standard addition technique as listed in Table 5. Accepted percentage recoveries and standard deviation values confirmed the validity of suggested methods and absence of any interference due to pharmaceutical formulation excipients.

**Table 5:** Determination of paracetamol, caffeine and drotaverine hydrochloride in their pharmaceutical formulation using the proposed chemometric methods and the results of standard addition technique

Pharmaceutical formulation	Compound	PCR	PLS	siPLS
		Found^a^ % ± SD		
Petro® tablets	PAR	104.37 ± 1.698	103.78 ± 2.125	104.05 ± 0.982
	CAF	94.67 ± 2.032	99.31 ± 1.833	98.21 ± 2.063
	DRO	96.85 ± 2.059	97.35 ± 1.268	103.00 ± 1.789
Standard addition	PAR	99.99 ± 1.342	100.51 ± 1.892	99.98 ± 1.698
	CAF	100.65 ± 1.768	99.18 ± 0.970	99.62 ± 0.589
	DRO	99.50 ± 1.137	100.22 ± 1.660	100.52 ± 1.288
	PAP	98.47 ± 0.686	99.82 ± 1.617	99.58 ± 1.838
	THEO	97.25 ± 0.922	98.99 ± 1.773	98.06 ± 1.007
	HVA	100.65 ± 1.768	99.98 ± 1.698	97.02 ± 1.917

^a^Average of five determinations

### Statistical comparison

The results gained from the assay of the studied pharmaceuticals by the suggested chemometric models and the reported HPLC method [19] in Petro® tablets were statistically compared. The calculated values of the t and F tests are found to be lower than their respective theoretical ones, indicating that there is no discernible difference between the developed methods and the reported one in regards of accuracy and precision [34]. Results are represented in Table 6. Furthermore, one-way analysis of variance (ANOVA) statistical test was conducted in order to compare the results obtained by the three developed approaches (PCR, PLS and siPLS), Additional file 1: Table S4. The obtained results confirm absence of any significant difference between the proposed methods as the calculated F is less than the critical one [34].

**Table 6:** Statistical analysis of the results obtained by the proposed chemometric methods and the reported method for the determination of paracetamol, caffeine and drotaverine hydrochloride in their pharmaceutical formulation

Item	PCR			PLS			siPLS			Reported method* [19]		
	PAR	CAF	DRO	PAR	CAF	DRO	PAR	CAF	DRO	PAR	CAF	DRO
Mean	104.37	94.67	96.85	103.78	99.31	97.35	104.05	98.21	103.00	102.17	97.2	98.34
SD	1.698	2.032	2.059	2.125	1.833	1.268	0.982	2.063	1.789	1.751	1.456	1.128
Variance	2.883	4.129	4.239	4.516	3.360	1.608	0.964	4.256	3.201	3.066	2.120	1.272
An	5	5	5	5	5	5	5	5	5	5	5	5
Student’s t-test (2.306)^**^	2.017	2.263	1.419	1.307	2.015	1.304	2.094	0.894	0.751			
F value (6.39)^**^	1.063	1.948	3.332	1.473	1.585	1.264	3.180	2.008	2.517			

^**^ Figures in parentheses are the corresponding tabulated values for t and F at p = 0.05

*HPLC method using C_8_ column with mobile phase composed of methanol: 0.02 M sodium dihydrogen phosphate (50:50, v/v) at a flow rate of 1.0 mL/min and UV detection wavelength at 220.0 nm

## Conclusion

Ultimately, this study has shown the utility of variable selection in resolving the difficulties of spectral overlapping. The suggested multivariate calibration methods have the merits of good accuracy, specificity, and reproducibility. These methods can be easily exploited in the routine analysis of the studied compounds in their pure powdered form as well as in pharmaceutical tablet formulation without interference that may come from impurities or excipients. PCR, PLS, and siPLS approaches have been found to be a realistic option for rapid analysis of the mixtures, with the advantages of being cost effective and time saving. They are also applicable in laboratories lacking sophisticated instruments as liquid chromatography ones. Comparatively to the applied PCR and PLS models, siPLS has reduced values of latent variables and root mean square error of prediction. siPLS model has a better performance when used to quantify PAR, CAF and DRO in existence of their corresponding impurities in synthetic laboratory mixtures and tablet dosage form.

### Supplementary Information

**Additional file 1: Figure S1.** Residual error values versus the true concentration for the six studied components using PCR chemometric method [X- axis is Concentration (µg/mL), Y-axis is Error = (theoretical concentration – found concentration)]. **Figure S2.** Residual error values versus the true concentration for the six studied components using PLS chemometric method [X- axis is Concentration (µg/mL), Y-axis is Error = (theoretical concentration – found concentration)]. **Figure S3.** Residual error values versus the true concentration for the six studied compounds using siPLS chemometric method [X- axis is Concentration (µg/mL), Y-axis is Error = (theoretical concentration – found concentration)]. **Table S1**. Determination of the studied components in laboratory prepared mixtures in the validation set by the proposed (PCR and PLS) chemometric methods. **Table S2.** Error associated with each compound using siPLS chemometric model. **Table S3.** Determination of the studied components in laboratory prepared mixtures in the validation set by the proposed siPLS chemometric method. **Table S4.** Results of One-Way ANOVA for comparison of the three proposed methods (PCR, PLS and siPLS).

## Data Availability

The data used and/or analyzed during this study are available from the corresponding author on a reasonable request.
