# Supplementary material for: Smart chemometrics-assisted spectrophotometric methods for efficient resolution and simultaneous determination of paracetamol, caffeine, drotaverine HCl along with three of their corresponding related impurities
Source: BMC Chem. 2023 Oct 5;17(1):133. doi: 10.1186/s13065-023-01036-8 (PMC10557158; doi:10.1186/s13065-023-01036-8)
Supplement: Supplementary file 1 — Additional file 1: Figure S1. Residual error values versus the true concentration for the six studied components using PCR chemometric method [X- axis is Concentration (µg/mL), Y-axis is Error = (theoretical concentration – found concentration)]. Figure S2. Residual error values versus the true concentration for the six studied components using PLS chemometric method [X- axis is Concentration (µg/mL), Y-axis is Error = (theoretical concentration – found concentration)]. Figure S3. Residual error values versus the true concentration for the six studied compounds using siPLS chemometric method [X- axis is Concentration (µg/mL), Y-axis is Error = (theoretical concentration – found concentration)]. Table S1. Determination of the studied components in laboratory prepared mixtures in the validation set by the proposed (PCR and PLS) chemometric methods. Table S2. Error associated with each compound using siPLS chemometric model. Table S3. Determination of the studied components in laboratory prepared mixtures in the validation set by the proposed siPLS chemometric method. Table S4. Results of One-Way ANOVA for comparison of the three proposed methods (PCR, PLS and siPLS). [file 13065_2023_1036_MOESM1_ESM.docx]

**Additional Information** for

**“Smart Chemometrics- assisted Spectrophotometric Methods for Efficient Resolution and Simultaneous Determination of Paracetamol, Caffeine, Drotaverine HCl along with Three of their Corresponding Related Impurities’’**

Samia A. Tawfik* ^1^, Maha A. Hegazy^1^, Nariman A. El-Ragehy^1^ and Ghada A. Sedik^1^

^1^ Department of Analytical Chemistry, Faculty of Pharmacy, Cairo University, Kasr El-Aini St., 11562, Cairo, Egypt

***Corresponding author**: Samia A. Tawfik

**E-mail address of corresponding author:** [**samia.ali@pharma.cu.edu.eg**](mailto:samia.ali@pharma.cu.edu.eg)

|  |  |
| --- | --- |
|  |  |
|  |  |

**Figure S1: Residual error values versus the true concentration for the six studied components using PCR chemometric method [X- axis is Concentration (µg/mL), Y-axis is Error = (theoretical concentration – found concentration)]**

|  |  |
| --- | --- |
|  |  |
|  |  |

**Figure S2: Residual error values versus the true concentration for the six studied components using PLS chemometric method [X- axis is Concentration (µg/mL), Y-axis is Error = (theoretical concentration – found concentration)]**

|  |  |
| --- | --- |
|  |  |
|  |  |

**Figure S3: Residual error values versus the true concentration for the six studied compounds using siPLS chemometric method [X- axis is Concentration (µg/mL), Y-axis is Error = (theoretical concentration – found concentration)]**

**Table S1: Determination of the studied components in laboratory prepared mixtures in the validation set by the proposed (PCR and PLS) chemometric methods**

| **Mixture no.** | **PCR** | | | | | | **PLS** | | | | | |
| --- | --- | --- | --- | --- | --- | --- | --- | --- | --- | --- | --- | --- |
|  | **Recovery%** | | | | | | | | | | | |
|  | **PAR** | **CAF** | **DRO** | **PAP** | **THEO** | **HVA** | **PAR** | **CAF** | **DRO** | **PAP** | **THEO** | **HVA** |
| **10** | 99.33 | 102.57 | 96.39 | 100.78 | 102.71 | 99.67 | 98.92 | 102.69 | 99.773 | 100.64 | 102.15 | 98.96 |
| **13** | 101.28 | 94.46 | 97.14 | 98.88 | 103.08 | 100.39 | 99.79 | 95.07 | 102.10 | 99.26 | 101.62 | 100.11 |
| **19** | 85.00 * | 98.27 | 106.34 | 102.25 | 97.06 | 97.07 | 88.12* | 98.47 | 103.00 | 103.17 | 96.71 | 98.25 |
| **20** | 98.34 | 98.846 | 102.50 | 97.47 | 99.86 | 100.34 | 100.08 | 98.72 | 100.32 | 97.36 | 101.29 | 100.92 |
| **23** | 100.86 | 103.43 | 117.63* | 97.05 | 100.26 | 100.11 | 101.82 | 103.38 | 98.99 | 97.26 | 101.68 | 101.61 |

* Rejected values according to Q rejection rule ^[34]^.

**Table S2: Error associated with each compound using siPLS chemometric model**

| **Studied compound** | **Error** |
| --- | --- |
| **PAR** | 1.3459 |
| **CAF** | 0.83153 |
| **DRO** | 0.76483 |
| **PAP** | 0.67595 |
| **THEO** | 0.61994 |
| **HVA** | 0.5582 |

**Table S3: Determination of the studied components in laboratory prepared mixtures in the validation set by the proposed siPLS chemometric method**

| **Mixture no.** | **siPLS** | | | | | |
| --- | --- | --- | --- | --- | --- | --- |
|  | **Recovery%** | | | | | |
|  | **PAR** | **CAF** | **DRO** | **PAP** | **THEO** | **HVA** |
| **10** | 99.55 | 100.2 | 101.88 | 97.06 | 100.4 | 101.55 |
| **13** | 101.94 | 97.36 | 99.92 | 98.52 | 99.4 | 98.93 |
| **19** | 90.62* | 97.77 | 100.69 | 101.03 | 99.96 | 100.09 |
| **20** | 101.11 | 96.88 | 102.82 | 98.63 | 101.71 | 100.36 |
| **23** | 100.03 | 97.94 | 100.65 | 98.03 | 101.33 | 100.73 |

* Rejected value according to Q rejection rule ^[34]^.

**Table S4**: **Results of One-Way ANOVA for comparison of the three proposed methods** (**PCR, PLS and siPLS)**

| **Studied compound** | **Source**  **of variation** | **Sum of squares (SS)** | **Degree of freedom (df)** | **Mean square (MS)** | **F-value** | **F-critical** |
| --- | --- | --- | --- | --- | --- | --- |
| **PAR** | **Between groups** | 1.056 | 2 | 0.528 | 0.352554 | 4.256495 |
|  | **Within groups** | 13.480 | 9 | 1.498 |  |  |
|  | **Total** | 14.536 | 11 |  |  |  |
| **CAF** | **Between groups** | 8.175 | 2 | 4.088 | 0.466465 | 3.885294 |
|  | **Within groups** | 105.153 | 12 | 8.763 |  |  |
|  | **Total** | 113.328 | 14 |  |  |  |
| **DRO** | **Between groups** | 0.824 | 2 | 0.412 | 0.054857 | 3.982298 |
|  | **Within groups** | 82.625 | 11 | 7.511 |  |  |
|  | **Total** | 83.449 | 13 |  |  |  |
| **PAP** | **Between groups** | 2.074 | 2 | 1.037 | 0.237071 | 3.885294 |
|  | **Within groups** | 52.490 | 12 | 4.374 |  |  |
|  | **Total** | 54.564 | 14 |  |  |  |
| **THEO** | **Between groups** | 0.045 | 2 | 0.028 | 0.005727 | 3.885294 |
|  | **Within groups** | 47.621 | 12 | 3.969 |  |  |
|  | **Total** | 47.666 | 14 |  |  |  |
| **HVA** | **Between groups** | 1.672 | 2 | 0.836 | 0.526258 | 3.885294 |
|  | **Within groups** | 19.06 | 12 | 1.588 |  |  |
|  | **Total** | 20.732 | 14 |  |  |  |

At the 0.05 level, the population means are not significantly different ^[34]^.
